# Tuberculous Cripples

**Published:** 1921-09-17

**Authors:** 


### The Hospital

The Workers' Newspaper of Administrative Medicine and Institutional Life, Administration, National Insurance and Health.

No. 1841, Vol. LXX. SATURDAY, SEPTEMBER 17, 1921. PRICE TWOPENCE

### TUBERCULOUS CRIPPLES.

The question of crippled children comes now to be an important item in any school medical officer's agenda list. Of course, one great source of crippling is bone and joint tuberculosis; we notice that in two excellent reports by, respectively, Dr. Joseph, of Warrington, and Dr. Daley, of Blackburn, tuberculosis accounts for most of the cases in the one, and stands second only to infantile paralysis in the other. Moveover, as is well known, the decline in phthisis of recent decades has not been shared by surgical tubercle. If, then, prevention has for some occult reason failed somewhat in this particular form of the disease, care and treatment of the actual patient becomes all the more urgent. By the very nature of the case it is always an important matter, because spinal or hip disease is a chronic ailment which, even if it spares life for years, stops a child's education, and is only too likely to make him unemployable as a manual worker.

In the past such cases generally used to be sent to the surgical wards of a general hospital, special institutions being very scarce. The demerits of this plan are obvious when we consider that general hospitals are far too crowded to keep in bone tuberculosis cases the great length of time necessary, as also that ambulant treatment is an impossibility, the first requisite being immobilisation. As Dr. Daley says—" When one considers the difficulties which beset poor mothers who are endeavouring to obtain treatment for these crippled children, it is a matter for surprise that so much has been done. It is not uncommon for mothers to have to carry the children at regular and fairly frequent intervals to the infirmary, or even take them to Liverpool or Manchester. The apparatus is costly, and requires renewal from time to time." It is to be feared that even yet this apology for proper care continues to some considerable extent, because, although special hospitals for surgical tubercle have become more numerous, still the few beds available for even a large town will soon be filled, and will very slowly indeed empty.

Again, the provision of large orthopædic hospitals has been urged, whither all crippled children, tuberculous and other, can be sent. There are many objections to this plan, practical and technical. To take the latter first—the treatment of tuberculosis differs from that of congenital deformity, &c., in necessitating open-air arrangements and heliotherapy. By mixing cases there is also some slight risk of infection, and a very real one of the imputation of infection. And practically there is no prospect at all for years of the money, or material, necessary for building. When schools are advertising in vain for asphalte with which to mend their playgrounds, and existing hospitals are closing wards, what chance for a large number of fresh structures? The idea is financially impossible. We are glad to see that Dr. Joseph in Section XV. of his study of the subject keeps the two things apart, postulating institutional accommodation for the tuberculous cases, and for the others a local orthopædic centre, with electrical and other apparatus and the methods usually employed for them.

But now that tuberculosis officers are to be found all over the country, these gentlemen should be encouraged to take an active part in the solution of the problem. In the best event, as things are and will be for a long time, there must be a waiting list of patients for treatment, and the waiting has perforce to be done at their homes. Now in the early stages of tuberculous crippling very simple measures, like extension, can do a great deal, especially in the alleviation of pain, the initiation of quiescence of the lesion, and the avoidance of deformity. Moreover, the refined technique of Berck-sur-Mer and other Continental places, so ably introduced to this country by Sir Henry Gauvain, can be studied in Hampshire, and, indeed, at one or two replicas elsewhere in this country. Experience has shown that the disease, when seen as early as the tuberculosis officer sees it, is very amenable to skilled treatment, even by an open window in a working-class dwelling; while even if such measures alone do not suffice, the long stay subsequently at a special hospital—and the length of time required is the one drawback to conservative treatment—will be very usefully curtailed. Dr. Daley might have added that the costly apparatus to which he refers is much inferior in efficiency to the celluloid splint. Moreover, both are useless without a preliminary period of rest, nearly always in bed. The time that inevitably has to be spent at home may be well exploited by the tuberculosis officer in getting the patient started in the right way of treatment. In not a few cases, indeed in most, he may, after a little experience, given keenness and a proper grounding in up-to-date methods, carry matters through by himself to a successful conclusion. As for the call on his time, well, with the extinction of the tuberculin craze, and the waning of the idea of contact examination being the key to prophylaxis, there should not be a great deal of difficulty in that direction.

